# Transversus abdominis plane block with liposomal bupivacaine compared to oral opioids alone for acute postoperative pain after laparoscopic hysterectomy for early endometrial cancer: a cost-effectiveness analysis

**DOI:** 10.1186/s40661-017-0048-7

**Published:** 2017-08-22

**Authors:** Brandon-Luke L. Seagle, Emily S. Miller, Anna E. Strohl, Anna Hoekstra, Shohreh Shahabi

**Affiliations:** 1 Department of Obstetrics and Gynecology, Prentice Women’s Hospital, Northwestern University, Feinberg School of Medicine, 250 E Superior Street, Suite 05-2168, Chicago, IL 60611 USA; 2 West Michigan Cancer Center and Western Michigan University, Homer Stryker School of Medicine, Kalamazoo, MI USA

**Keywords:** Endometrial cancer, Pain, Analgesia, Bupivacaine, Transversus abdominis plane block

## Abstract

**Background:**

To determine the cost-effectiveness of transversus abdominis plane block with liposomal bupivacaine (TAP) compared to oral opioids alone for acute postoperative pain after laparoscopic hysterectomy for early endometrial cancer.

**Methods:**

A cost-effectiveness analysis using a decision tree structure with a 30.5 day time-horizon was used to calculate incremental cost-effectiveness ratio (ICER) values per quality-adjusted life-year (QALY). Base-case costs, probabilities, and QALY values were identified from recently published all-payer national database studies, 2017 Medicare fee-schedules, randomized trials, institutional case series, or assumed, when published values were not available. One-way, two-way and multiple probabilistic sensitivity analyses were performed.

**Results:**

The TAP strategy dominated the oral opioid-only strategy, with decreased costs and increased effectiveness. Specifically, the TAP strategy saved $235.90 under the base-case assumptions. Threshold analyses demonstrated that if the relative same-day discharge probability was ≥ 12% higher in the TAP group, then TAP was cost-saving over oral opioids-alone. Similarly, TAP was cost-saving whenever the costs saved by same-day discharge compared to admission were ≥ $1115.22. Cost-effectiveness of the TAP strategy was highly robust of a variety of sensitivity analyses.

**Conclusions:**

TAP with liposomal bupivacaine was robustly cost-effective at conventional willingness-to-pay thresholds. Further, TAP was cost-saving compared to opioids-only when the same-day discharge rate among TAP users was greater than among opioid-only users.

**Electronic supplementary material:**

The online version of this article (doi:10.1186/s40661-017-0048-7) contains supplementary material, which is available to authorized users.

## Background

Enhanced recovery protocols have become the standard-of-care for gynecologic oncology surgery [1, 2]. These comprehensive protocols result in shorter lengths of stay, fewer complications, earlier return of bowel function, and increased patient satisfaction [1, 2]. Optimal analgesia for acute postoperative pain requires a multi-class medication strategy with the goal of opioid minimization [1, 2]. Epidural and patient-controlled analgesia (PCA) have lost favor in many practices given immediate initiation of oral intake and the concurrent goals of early ambulation and opioid minimization in the early postoperative period. Much of the experience supporting a potential benefit of either incisional or regional injection of long-acting local analgesics such as liposomal bupivacaine was not published at the time of development of the ERAS® Society guidelines [1, 2].

Recently, the Mayo clinic reported a series of gynecologic oncology patients who received surgeon-injected regular or liposomal bupivacaine at the laparotomy incision [3]. Women who received liposomal bupivacaine used less rescue intravenous or PCA opioids [3]. There was no difference in total pharmacy costs despite the higher cost of liposomal bupivacaine [3]. Women who received liposomal bupivacaine also had decreased nausea and ileus [3]. Because the innervation of the anterior abdominal wall anatomically travels from lateral to medial, and laparoscopy requires placement of laterally-located port sites, injection of liposomal bupivacaine by bilateral transversus abdominis plane (TAP) block may be particularly useful for women undergoing laparoscopic or robotic hysterectomy.

Anesthesiologists at one of the author’s hospitals routinely give TAP blocks with liposomal bupivacaine preoperatively to women undergoing laparoscopic or robotic surgery. This quality-improvement practice facilitated an 86% rate of same-day discharge (SDD) among women undergoing surgical staging for gynecologic malignancies in the last two-years. High SDD rates were also reported by a trial of women undergoing robotic hysterectomy and randomized to TAP with regular versus liposomal bupivacaine [4]. 60.7% (17/28) of women who received liposomal bupivacaine had same-day discharge [4]. Women who received liposomal bupivacaine had decreased pain scores throughout their first 72-h after surgery, significantly decreased opioid use, and less nausea and vomiting [4]. We believe that presumptions about higher costs and, in some systems, pharmacy formulary decisions against liposomal bupivacaine, have limited access to TAP blocks with liposomal bupivacaine. To address this concern, we performed a cost-effectiveness analysis of TAP with liposomal bupivacaine versus routine oral opioid-only analgesia for women undergoing laparoscopic hysterectomy for early endometrial cancer.

## Methods

### Decision tree

A decision tree was built to evaluate the cost-effectiveness of TAP block with liposomal bupivacaine versus routine oral opioid-only postoperative analgesia among women undergoing laparoscopic hysterectomy for early endometrial cancer (Additional files 1, 2 and 3: Figures S1–S3). The model was built from a healthcare system perspective, meaning that costs to the healthcare system but not greater societal costs were included. Incremental cost-effectiveness ratio (ICER) values were calculated in terms of costs per quality-adjusted life-year (QALY). An ICER value of < 50,000/QALY was considered cost-effective.

The decision node of the model was split by TAP with liposomal bupivacaine versus no TAP. In both arms of the decision tree women had a chance of SDD or admission, a chance of readmission or no readmission within 30 days, and a chance of opioid use or no opioid use. Downstream of opioid use, there was a complication subtree including costs and probabilities related to narcotic-use for aspiration and ileus. In the TAP arm, possibilities of a TAP procedure complication or liposomal bupivacaine adverse event were additionally modeled. Any patient who experienced either a TAP procedure complication or bupivacaine adverse event was assumed to be admitted. Probabilities reflecting the chances of overall 30-day mortality and of a composite complication (most commonly urinary tract infection) for women undergoing laparoscopic hysterectomy were included as chance events throughout both arms. Additional probabilities of death were modeled specifically for a bupivacaine adverse event, aspiration, or bowel perforation, which was assumed to be a very rare complication only in the postoperative ileus population. Finally, among women who used opioids, the 30-day mortality rate was increased by the prescription opioid fatal overdose risk of the population. At the end of model, each patient was either alive without complications, alive with complications, alive after perforation, alive after perforation and additional complications, or deceased. Model building and implementation, statistical analyses and figure creation were performed with TreeAge Pro 2016 (TreeAge Software Inc., Williamstown, MA).

The model operates under several additional noteworthy assumptions. First is the assumption that SDD is typically higher among women who have a TAP block than women who receive only oral opioids. This assumption is based on our experience and also the published SDD rates, decreased pain scores, better 72-h pain control, and decreased postoperative nausea and vomiting associated with use of liposomal bupivacaine for acute postoperative pain reported by previous studies [3, 4]. Since the major discharge criteria for SDD are adequate pain control and ability to tolerate oral analgesics and anti-emetics, if needed, this assumption is reasonable. Differences in opioid use were modeled as use or non-use rather than by quantity of use, given limitations of the existing literature. Further, since the serious narcotic-associated complications are uncommon, alternative modeling of these parameters would not change the statistical inferences resulting from this cost-effectiveness analysis. Complications were allowed even among women who were not readmitted, so as to not bias the model toward TAP block with its associated higher SDD probability, since readmission has been observed to be less common among women who had SDD. The analysis reasonably assumes no systematic differences in surgical procedures or oncologic outcomes among women who receive TAP versus opioid-only analgesia. The model used a short time-horizon of only 30.5 days as outcome differences related to strategy for postoperative pain management are assumed to be none by or before 30 days after surgery. Finally, the model assumes that anesthesiology personal are readily available to place TAP blocks, and that TAP blocks are placed in pre-operative holding before women enter the operating room, which is our routine practice. Therefore, there are no systematic differences in operating room time, or time under general anesthesia, among women who do versus do not receive TAP blocks.

### Costs

Cost data are shown in Table 1. Drug prices were the average wholesale prices, updated monthly, as referenced on UpToDate® drug information pages. Liposomal bupivacaine administration was modeled as typical TAP block use of a single vial of Exparel®, base-case cost of $204. The wholesale acquisition cost has been reported to be as low as $14.25 per vial [5]. Northwestern Memorial Hospital’s pharmacy has a hospital cost of $315 per vial. Prophylactic treatment for uncomplicated aspiration was presumed to be ampicillin-clavulanate 875–125 mg orally twice daily for 3 days. The online 2017 physician fee schedule facility limiting charges from the Centers for Medicare & Medicaid Services were used for reimbursement costs based on CPT codes. TAP procedure cost was per CPT code 64488 for bilateral injection. Cost of a serious bupivacaine adverse event was considered the cost of a cardiopulmonary resuscitation using CPT code 92950. Total cost for surgical admission, including costs of complications, were considered the observed costs for laparoscopic hysterectomy from Wright et al. using the nationally-representative all-payer Premier database [6]. Costs for same-day surgery were modeled as the costs from Wright et al. minus $2400, the average cost per inpatient day in the United States among nonprofit hospitals [7]. This assumes that admitted patients are typically discharge home on post-operative day 1, although some patients stay longer [7]. Costs of additional complications were considered the cost to treat a urinary tract infection, the most common complication after laparoscopic hysterectomy, with trimethoprim-sulphamethoxazole double-strength orally twice daily for 3 days. Costs of postoperative ileus were from Asgeirsson et al. [8]. Costs of routine opioid prescription were considered oxycodone 5 mg × 30 tablates. Costs of nonfatal bowel perforation were from Cohn et al. [9]. General readmission costs were used similarly as by Seagle et al. [10]. Cost of a serious TAP block complication was considered the cost of a computed tomography scan of the abdomen and pelvis (CPT 74177) to diagnose the complication [11].

**Table 1:** Cost estimates

Cost	Base-case ($)	Range (S)	Reference
30 day mortality	5000	2000–10,000	Assumed
Aspiration	30	0–100	UpToDate
Liposomal bupivacaine	204	50–350	UpToDate
Liposomal bupivacaine adverse event	209.76	100–300	CMS
Composite complication	18.72	10–30	UpToDate
Postoperative ileus	8296	1000–15,000	8
Oral opioids	16.23	10–25	UpToDate
Nonfatal bowel perforation	138,000	20,000–200,000	9
Readmission	10,000	5000–15,000	10
Laparoscopic hysterectomy	6679	5197–8673	6
Cost reduction for same-day discharge	2400	1000–4000	7
TAP procedure	83.12	75–100	CMS
TAP complication	315.70	200–450	CMS

Abbreviations: *CMS* Centers for Medicare & Medicaid Services

### Effectiveness

Effectiveness was framed in terms of QALY values. Alive states were given baseline QALY values of 0.85. This calculation accounted for 70% of women with clinically early endometrial cancer being surgically staged as stage I and therefore without evidence of disease after surgery. These women have a high expectation of cure. In contrast, 30% were considered to be alive with cancer or with pathologic high-risk factors requiring adjuvant therapy, and were assigned a QALY of 0.5 [12]. Having experienced a complication is assumed to further decrease the QALY value by 30% of baseline, except for having survived a bowel perforation, assumed to decrease the QALY by 80% of baseline during the 30 day study period. A randomized trial in laparoscopic hysterectomy reported a 10% increase in patient satisfaction scores comparing TAP with liposomal bupivacaine to TAP with regular bupivacaine [4]. While QALY values and patient satisfaction are not the same, we sought to incorporate this finding by assigning surviving women who underwent TAP with liposomal bupivacaine, compared to oral opioids-only, an additional 0.1 QALY value. Finally, all QALY values were adjusted to 30.5 day values by multiplication by 30.5/365.

### Probabilities

Probabilities were assigned as listed in Table 2. Composite complication probabilities for surgery were considered to occur with a 9.0% probability for the base-case and varied from 5.0–12.0% based on Wright et al. (laparoscopic hysterectomy for benign disease from the Premier database) and Scalici et al. (laparoscopic staging for endometrial cancer from the ACS-NSQIP database) [6, 13]. Probability of dying from bowel perforation was considered 25% from Cohn et al. [9]. The 30-day mortality probability for laparoscopic endometrial cancer surgery was 0.14% from Gildea et al. [14]. For women using opioids, the 30-day mortality probability was increased by 100/10,000,000, the population probability of prescription opioid fatal overdose [15]. Probabilities of postoperative ileus with or without opioid use among patients who had abdominal surgery were from Goettesch et al. [16]. Probabilities of an aspiration event without or with ileus were assumed to be 1 and 5% due to lack of literature references for these values. There are no estimates in the English language literature for some less common risks, which were therefore assumed (Table 2). For instance, there were no cases of local anesthetic complications reported with use in TAP block, per a published literature review [11]. There are also no case reports of death from a TAP block procedure complication [11]. However, we estimated a low-risk of serious liposomal bupivacaine adverse event based on tachycardia or bradycardia probabilities [17]. Bowel perforation as a very rare complication of postoperative ileus or obstruction was modeled based on case reports and series with an assumed base-case probability of 1/1000 cases of ileus [18]. Probability of readmission within 30 days was 2.5% for women who had SDD and 4.0% for admitted patients based on an ACS-NSQIP study [19]. Probability of SDD among women who got TAP with liposomal bupivacaine was estimated at 90% for the base-case model. The base-case probability of SDD was considered to be 0.78 × the SDD probability of women who underwent TAP, based on SDD proportions without TAP blocks reported as high as 70–75% in series where SDD was prioritized [20, 21]. These high SDD rates, however, are far from typical, with national surveillance database estimates being about 8–9% [19]. Finally, use of postoperative opioids among women given a TAP block is modeled as 50% of the probability of opioid use in the opioid-only group [4].

**Table 2:** Probability estimates

Probability	Base value (%)	Range (%)	Reference
30 day mortality	0.14	0.02–1.0	14
Fatal opioid overdose	0.001	0–0.002	15
Postoperative ileus without opioids	0.17	0–0.34	16
Postoperative ileus with opioids	1.43	0.43–2.43	16
Aspiration with ileus	5	0–10	Assumed
Aspiration without ileus	1	0–2	Assumed
Death by aspiration	1	0–2	Assumed
Bowel perforation with ileus	0.1	0–0.2	Assumed
Death from bowel perforation	25	10–40	9
Composite complication	9	5–12	6, 13
Opioid use without TAP block	95	90–100	Assumed
Fractional opioid difference with TAP	50	100–30	4
Same-day discharge (SDD), TAP	90	80–99	4
Fractional SDD difference, no TAP	78	90–50	19, 21
Readmission after SDD, TAP	2.5	1–5	19
Readmission after SDD, no TAP	2.5	1–5	19
Readmission after admission, TAP	4.0	1–5	19
Readmission after admission, no TAP	4.0	1–5	19
TAP procedure complication	0.1	0–0.2	11
Bupivacaine adverse event	1.5	0–3	11
Death by bupivacaine adverse events	0.1	0–0.2	Assumed

### Sensitivity analyses

One-way sensitivity analyses were performed and reported as tornado diagrams to evaluate how potential variation in each model parameter impacts the calculated ICER value estimates. Cost and probability estimates were varied across the ranges shown in Tables 1 and 2 for one-way sensitivity analyses and for probabilistic sensitivity analyses, using triangle distributions. A two-way sensitivity analysis was performed on the SDD probability in the TAP group and the proportional difference in SDD among the oral opioid group to better describe the results in terms of these important parameters. The probabilistic sensitivity analysis (1000 parameter re-samplings) was used to generate 95% confidence intervals for the base-case model ICER value estimate. Additionally, cost-effectiveness acceptability curves were plotted. Finally, the probabilistic sensitivity analyses were repeated with difference specifications of the base-case estimates, as indicated with Results, to test how our statistical inferences may vary under different major assumptions of the model.

## Results

### Base-case analysis

The TAP block strategy dominated the oral opioid-only strategy, with negative ICER values indicating decreased costs and increased effectiveness associated with TAP block use (Table 3). Specifically, the TAP block strategy was cost-saving of $235.90 under the base-case assumptions. Due to the short time-horizon of the analysis, the absolute QALY difference is low. If the increased QALY associated with patient satisfaction was assumed to be 0 rather than 0.1 in the base-case, TAP block was still cost-saving, as it had decreased cost at the same effectiveness.

**Table 3:** Cost effectiveness of TAP with liposomal bupivacaine compared to oral opioids

Regimen	Cost ($)	Incremental cost ($)	30 day QALY	Incremental	ICER ($/QALY)	95% CI ($/QALY)
TAP	5193.30	−235.90	0.08	0.01	−28,274.70	−10,177.86, −112,226.67
No TAP	5429.20		0.07			

Abbreviations: *TAP* transverses abdominis plane block, *QALY* quality-adjusted life-year, *ICER* incremental cost effectiveness ratio, *CI* confidence interval

### One-way sensitivity analyses

**Fig. 1:**
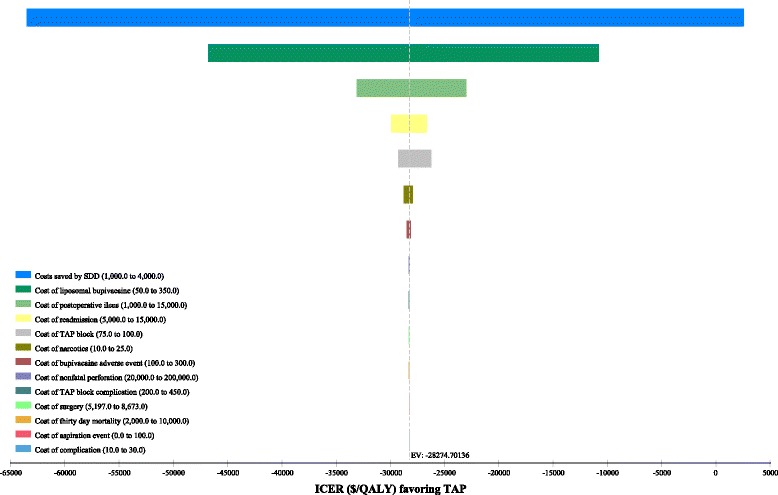
Tornado diagram for one-way sensitivity analyses of cost estimates

**Fig. 2:**
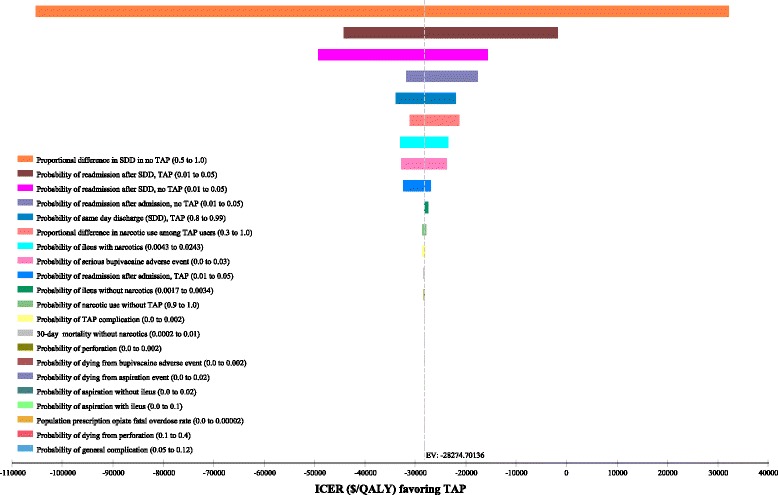
Tornado diagram for one-way sensitivity analyses of probability estimates

Tornado plots for one-way sensitivity analyses for all cost and probability estimates are shown in Figs. 1 and 2. For costs, ICER values were most sensitive to costs saved by SDD and costs of bupivacaine (Fig. 1). For probabilities, ICER values were most sensitive to the proportional difference in SDD in the no TAP group (Fig. 2). However, when this difference was 1.0, meaning that both strategies had an equal 90% SDD rate, the ICER for TAP was still cost-effective compared to a conventional willingness-to-pay of $50,000/QALY (Fig. 2).

Threshold analyses for when TAP block would no longer be cost-saving demonstrated that if the SDD probability was ≥12% higher (relative difference, not absolute difference) in the TAP group than the oral opioid group, then TAP was cost-saving over oral opioids-alone. Similarly, TAP was cost-saving when the costs saved by SDD were ≥ $1115.22 compared to admission, which would be true even in states with the lowest daily hospitalization costs [7].

### Two-way sensitivity analysis

**Fig. 3:**
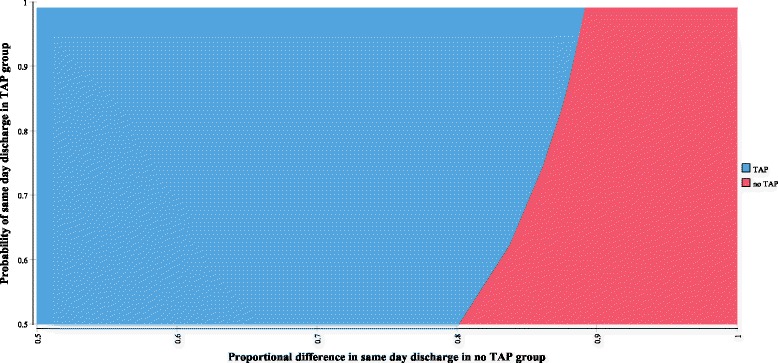
Two-way sensitivity analysis for TAP versus oral opioid-only strategies. Legend: *Blue area* is when the TAP strategy is cost-saving and *red* area is when oral opioid-only strategy is cost-saving

A two-way sensitivity analysis for the probability of SDD in the TAP group (range 50%–99%) versus the proportional difference in SDD in the oral opioid group (range 0.50–1.00), with willingness-to-pay set to $0/QALY to indicate the cost-saving strategy, showed that even at a low 50% SDD probability in the TAP group, TAP was cost-saving compared to oral opioids-only so long as the absolute SDD probability was ≤ 40% in the oral opioid-only group (Fig. 3).

### Probabilistic sensitivity analyses

**Fig. 4:**
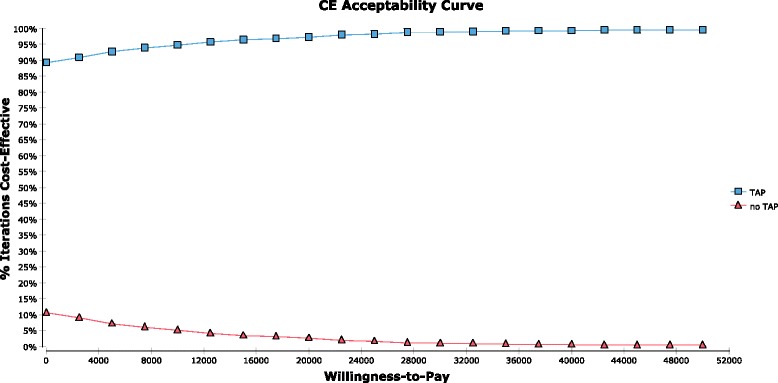
Cost-effectiveness acceptability curves for TAP versus no-TAP strategies from probabilistic sensitivity analyses

In the initial probabilistic sensitivity analysis, TAP was cost-effective compared to a conventional willingness-to-pay of $50,000/QALY in 99.5% of simulations, and cost-saving with ICER < $0/QALY in 89.3% of simulations. Cost acceptability curves for each strategy are shown in Fig. 4.

Additional probabilistic sensitivity analyses were performed under different major assumptions of the model and demonstrated highly robust results. First, if the oral opioid group was allowed to have an equal probability of SDD as the TAP group in sensitivity analyses, then TAP was cost-saving in 80.5% of simulations. Second, if the base-case probabilities of SDD were assumed to be 90% in both the TAP and oral opioid groups, with the opioid group allowed to have equal probability of SDD as the TAP group in sensitivity analyses, TAP was still cost-saving in 65.4% of simulations and cost-effective in 96.7% of simulations. Third, if the cost of liposomal bupivacaine was changed to the price of $315 (Northwestern Memorial Hospital’s cost to purchase) with range of $200–400 per vial, TAP was cost-saving in 76.9% of simulations and cost-effective in 98.8% of simulations. Fourth, if the probabilities of readmission were assumed to be equally low at 2.5% (range 1–5%) in both groups, TAP remained cost-saving in 89% of simulations. Finally, if the QALY increase associated with TAP use was removed from the model, TAP remained cost-saving in 90.2%.

## Discussion

Compared to the routine practice of prescribing oral opioids after laparoscopic hysterectomy, TAP block with liposomal bupivacaine is robustly cost-effective and in most scenarios is also cost-saving. This finding is robust to many alternative specifications of base-case parameters, and to changing their likeliest values used for probabilistic sensitivity analyses. Therefore, even if the major model assumptions are not agreed upon by all readers or representative of some practices (for instance, those with lower SDD rates), the main results are robust to alternatives in model assumptions. The cost-effectiveness of the TAP strategy was most sensitive to the difference in SDD probability between women who received TAP versus oral opioids-only. The two-way sensitivity analysis showed that TAP was cost-saving even at a low SDD probability of 50% among TAP users, with a 20% decreased relative SDD probability among oral opioid-only users, giving them an also low SDD probability of 40%.

Nationally, the SDD rate after laparoscopic surgical staging remains low at 8–9%, despite an only 2.5% readmission probability after SDD [19]. Some institutions have reported high (70–75%) SDD rates without use of TAP blocks [20, 21]. The randomized trial of TAP with regular versus liposomal bupivacaine had > 60% SDD in the liposomal bupivacaine arm [4]. In our experience, high SDD rates are routinely achieved using TAP with liposomal bupivacaine. The cost-effectiveness analysis here confirms that TAP block with the more expensive but longer-acting liposomal formulation of bupivacaine is typically cost-saving.

Achieving routine SDD for women undergoing laparoscopic or robotically-assisted laparoscopic surgical staging for early endometrial cancer offers an opportunity for healthcare system cost-savings, with improved postoperative recovery and patient satisfaction. Implementing routine SDD requires system and practice-wide commitments, beginning with preoperative patient counselling to expect outpatient surgery. Operative times for surgical procedures need to be consistently efficient, which can be more difficult to achieve in training environments with rotating surgical teams. Multidisciplinary support for SDD, with collaboration from operating room and recovery room anesthesiologists and nurses, is critical to ensure the success of SDD protocols. In fact, much of the success of a SDD program likely depends on anesthesiology and nursing practices.

In addition to limitations related to the base-case model assumptions, limitations of this study include that not all possible specific complications were modeled. For instance, postoperative pneumonia and venous thromboembolism were not specifically considered. However, these complications would presumably occur more often among women who were admitted to the hospital, favoring the TAP block strategy. Furthermore, cost estimates used for the surgical admission were total inpatient cost estimates including costs related to the full-spectrum of complications experienced, based on a nationally-representative all-payer database analysis, and therefore include costs of other additional complications. Also, because major complications are uncommon, consistent with the one-way sensitivity analysis results, the results are not sensitive to major complication costs. Therefore, disagreement about assumptions of modeling uncommon major complications, leading to alternative modeling of complications, would not meaningfully change the result that the TAP strategy is robustly cost-effective and often cost-saving. Surgical procedure costs used pertained to women undergoing hysterectomy for benign disease rather than for cancer. If higher surgical admission costs specifically for cancer or for robotic hysterectomy were used, the results would be very similar because the difference in SDD probabilities and cost-saving associated with SDD would be the same. These SDD parameters are the most important factors for determining the relative cost-effectiveness and the cost-savings of the TAP strategy. As for the potential concerns about assumptions of effectiveness estimates (QALY values) used here, it should be noted that no case report of death resulting from a TAP block with a local analgesic has been reported, and therefore the probability of death due to the TAP strategy that was assumed could arguably be removed from the model. This would allow us to transform the analysis to a cost-minimization, without consideration of effectiveness estimates or alternative time-horizons. A cost-minimization would show the TAP strategy to be most often cost-saving, consistent with the cost-effectiveness analysis results. Finally, use of non-opioid analgesic agents was assumed to be similar among the model arms in this decision analysis and was therefore not modeled. Given modern enhanced recovery protocols, it is likely that any institution using TAP blocks routinely also uses multi-modal oral analgesia strategies as part of good practice.

## Conclusions

TAP with liposomal bupivacaine is robustly cost-effective and most often cost-saving. This finding should be considered by hospital administrators and pharmacists when making their pharmacy formulary decisions. Anesthesiologists and surgeons with concerns about seemingly increased costs related to use of liposomal bupivacaine should be reassured that so long as SDD is routine for women with laparoscopic/robotic surgery, then the TAP strategy is cost-saving. Furthermore, cost-savings associated with use of liposomal bupivacaine may not hinge entirely on superior SDD probabilities among women who receive TAP. For instance, in our probabilistic sensitivity analysis that assumed high and equal 90% SDD probabilities in both the TAP and oral opioid-only groups, TAP remained cost-saving in 65% of simulations. Use of bilateral TAP blocks with liposomal bupivacaine as an analgesic strategy to facilitate SDD is most often cost-saving, and nearly always cost-effective compared to conventional willingness-to-pay thresholds.

## Additional files

Additional file 1: Figure S1. TAP strategy arm of the decision tree. (TIFF 16314 kb)

Additional file 2: Figure S2. No-TAP strategy arm of the decision tree. (TIFF 14084 kb)

Additional file 3: Figure S3. Complication subtree of the decision tree. (TIFF 18809 kb)

## Abbreviations

ICER: Incremental cost-effectiveness ratio
PCA: Patient-controlled analgesia
QALY: Quality-adjusted life-year
SDD: Same-day discharge
TAP: Transversus abdominis plane block
