# Supplementary figures and images for: Transversus abdominis plane block with liposomal bupivacaine compared to oral opioids alone for acute postoperative pain after laparoscopic hysterectomy for early endometrial cancer: a cost-effectiveness analysis
Source: Gynecol Oncol Res Pract. 2017 Aug 22;4:12. doi: 10.1186/s40661-017-0048-7 (PMC5567769; doi:10.1186/s40661-017-0048-7)

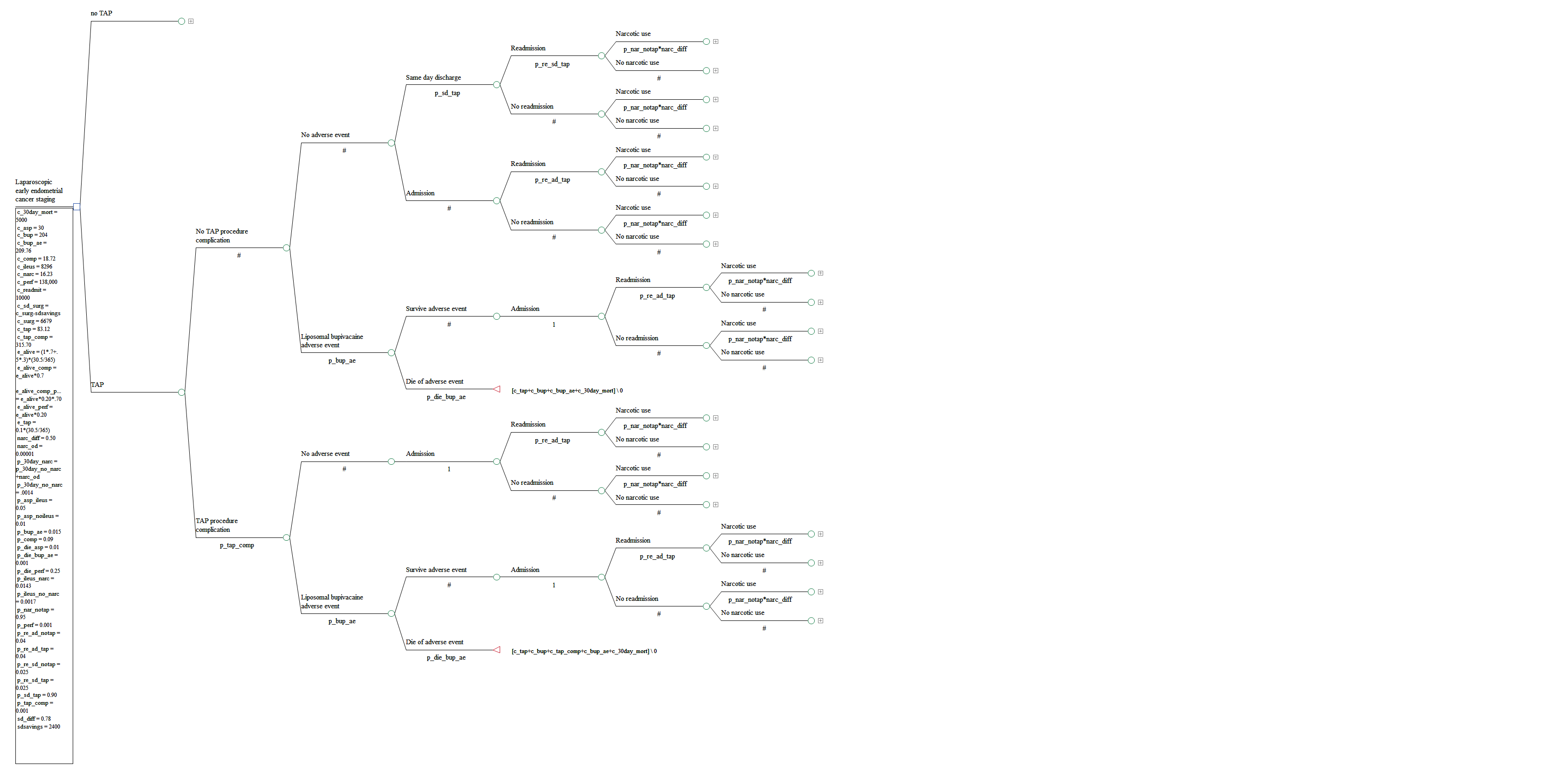

Supplement: Supplementary file 1 — TAP strategy arm of the decision tree. (TIFF 16314 kb) [file 40661_2017_48_MOESM1_ESM.tiff]

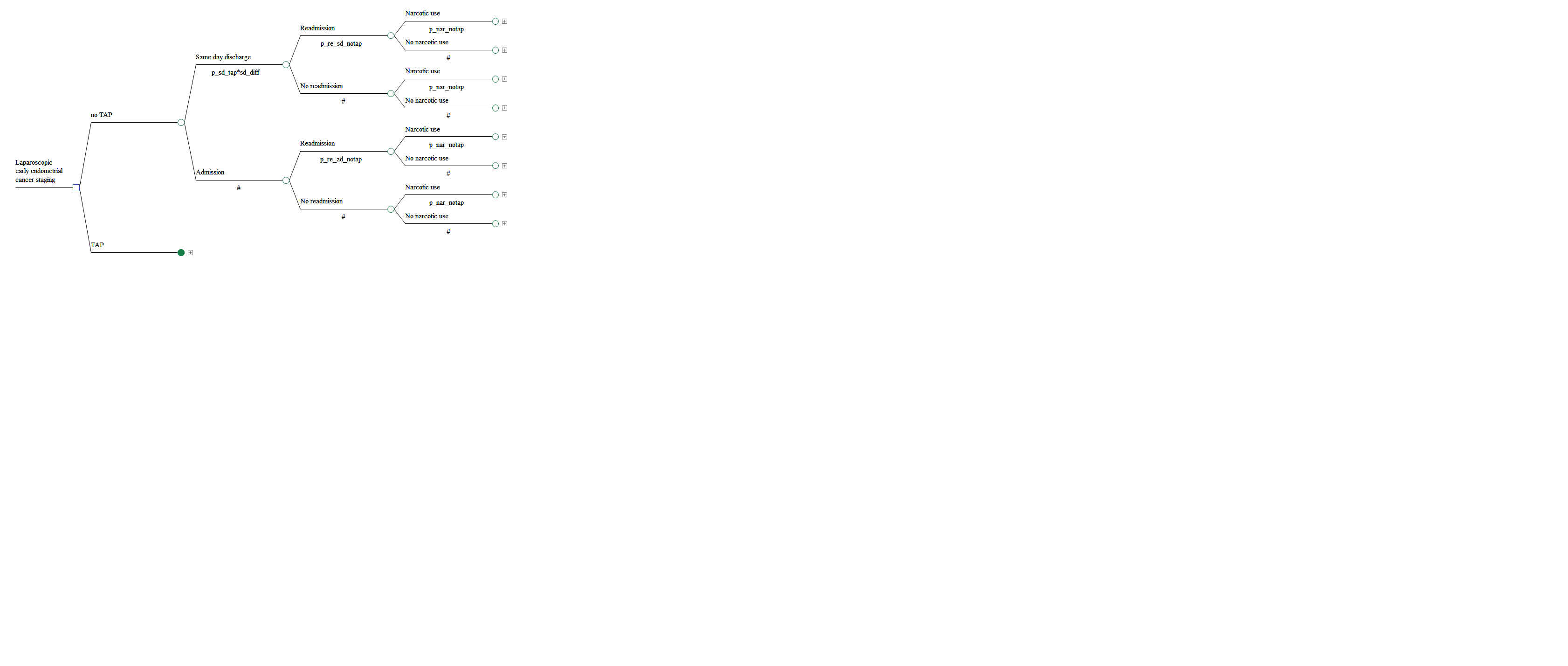

Supplement: Supplementary file 2 — No-TAP strategy arm of the decision tree. (TIFF 14084 kb) [file 40661_2017_48_MOESM2_ESM.tiff]

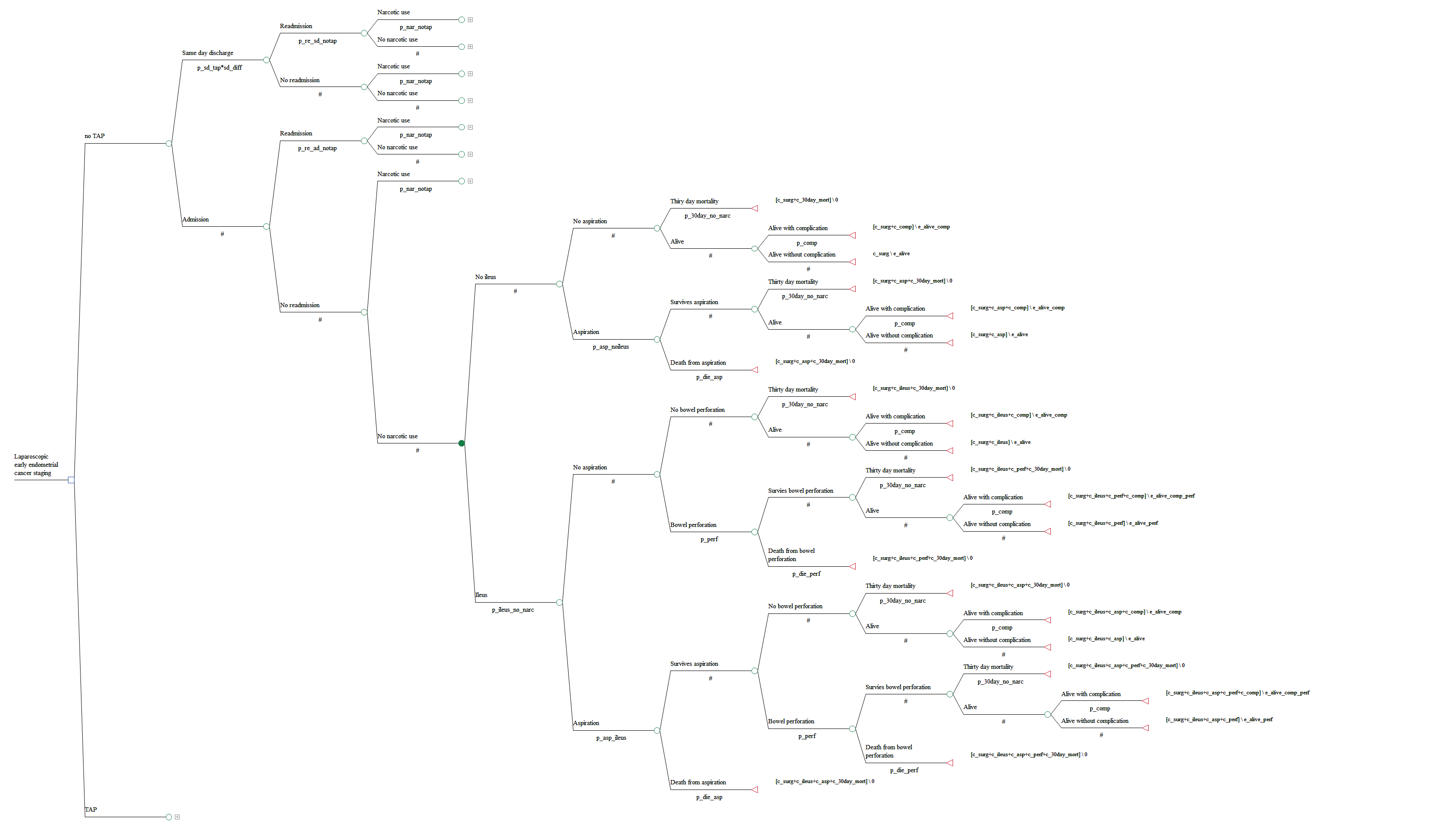

Supplement: Supplementary file 3 — Complication subtree of the decision tree. (TIFF 18809 kb) [file 40661_2017_48_MOESM3_ESM.tiff]
